# Chronic Aural Catarrh

**Published:** 1877-11

**Authors:** C. A. Simpson

**Affiliations:** Atlanta, Ga.


ATLANTA

MEDICAL AND SURGICAL JOURNAL

VOL. XV.] NOVEMBER—1877. [NO. 8

### Original Communications.

### CHRONIC AURAL CATARRH.

A PAPER READ BEFORE THE AOADEMY OF MEDICINE, OCT. 30, 1877.

BY C. A. SIMPSON, M.D., ATLANTA.

*Mr. President:*

The subject I have chosen for this paper is chronic aural catarrh. The seat of this annoying trouble is the mucous membrane of the cavity of the tympanum and eustachian tube. Of all the tissues of the body, the mucous membrane, from its vascularity, its exposed situation to various kinds of irritants, and its peculiar susceptibility to irritating influences, is most frequently affected with acute and chronic inflammation. The mucous membrane of the eustachian tube and middle ear being a continuation of that of the pharynx, and from the readiness with which inflammations extend along mucous tracts, the pharynx is the starting point in most cases of inflammation of the tubes and middle ear.

Two varieties of catarrh are recognized—sclerosis, or dry catarrh, in which the membrane has become thickened and inelastic, and moist catarrh, in which there is a congestive swelling, with increased secretion of the mucous membrane. Catarrh of the tube is described separately from catarrh of the middle ear, but in practice it is not always possible to make this distinction, as they are both so frequently affected at the same time.

Chronic aural catarrh is by far the most frequent cause of deafness for which the physician is consulted—almost every patient presenting himself with a history of gradually increasing deafness, beginning he hardly knows where, and with little pain, except when "he takes fresh cold and is troubled with chronic aural catarrh." The two other most frequent causes of deafness are impacted wax and acute inflammations. The acute inflammations (suppuration) are much more painful than chronic aural catarrh, while neither in it, or in the case of impacted wax, is there the history of gradually increasing deafness. Deafness from impaction of wax, if in only one ear, is frequently discovered suddenly, and probably accidentally.

A patient, consulting me for deafness in his right ear, stated that he was not aware that he was deaf in the right ear until he happened to close the left. Upon examination of the ear, I found it filled with wax, after the removal of which, and inflating the middle ear a few times, it was completely relieved.

In most cases of chronic aural catarrh, the patient seeks relief simply for the deafness, in one ear or both, though some are greatly annoyed by the noise in the ears, and are thankful if they can get relief from that alone.

There is a history of frequent colds in the head, and sore throat, and after each attack the hearing is worse. The mucous membrane of the pharynx is congested, and secretes a thick, glairy mucus.

Besides the symptoms mentioned, the diagnosis of chronic aural catarrh is made from the appearance and position of the membrana tympani and condition of the eustachian tube. The membrana tympani can be readily examined by means of a concave mirror, two or three inches in diameter, with a focal distance of five to six inches, and an ear speculum, either of silver or hard rubber. The best position for the examination is for the patient to be seated opposite, and with the side to, a window, while the examiner is seated on the other side, and examines the ear with reflected light. By drawing the ear upwards and outwards with the left hand, to straighten the canal, the speculum is readily introduced with the right. Now, by holding the speculum in position with the thumb and first finger of the left hand, and pulling the ear upwards and outwards with the second and third fingers, the light from the window is readily thrown into the ear, so as to illuminate the drum.

The normal color of the membrana tympani, as described by Politzer, is a "neutral gray, with the addition of a weak tint of violet and light brownish yellow." There is, however, a considerable variation of this neutral gray in the membrana tympani of those whose hearing is normal. The drum is translucent, and the normal gray, which is partly due to light passing through the drum, may be changed to a darker gray, with more or less mixture of yellow, due to thickening of the drum. The drum is usually drawn far inward, and has lost its brilliancy, and the normal triangular light spot may be cut up into several pieces, or reduced to a fine line or point.

In persons with a normal degree of hearing, the appearance of the drum varies considerably, and you may see the light spot vary greatly in size. The light spot depending upon the position of the drum, any variation in its normal degree of concavity produces a difference in the size or shape. When the drum is drawn very far inwards, it becomes a fine point or line.

An important means of diagnosis, as well as treatment, of affections of the middle ear, is by air forced through the eustachian tubes. The methods used are the Valsalvian and Politzer's, with or without the eustachian catheter.

The Valsalvian method consists in the patient closing the nose and mouth and expiring forcibly, when the air rushes along the tubes to the ear. In Politzer's method, a rubber air bag, with a nozzle attached, is used. The patient takes a mouthful of water in the mouth, the nozzle is inserted in the nostril, and the nostril firmly closed around it, so as to prevent the exit of air, the patient is told to swallow, and at the instant of swallowing, the air-bag is forcibly pressed with the right hand. The act of swallowing opens the mouths of the eustachian tubes, and the air is driven to the middle ear.

The catheter is a very important means of diagnosis and treatment, and, in most cases, is easily introduced. The patient should be seated opposite a window while the operator stands. While the left hand is on the back of the head so as to steady it, with the right the curved point of the catheter is inserted into the inferior meatus of the nose, the catheter being held in a vertical position; it is then raised to a horizontal position and pushed along the floor of the nasal cavity, until the point touches the posterior wall of the pharynx; the instrument is then drawn one-third to three-fourths of an inch backwards, the outer end slightly elevated and the point turned upwards and outwards towards the ear, when it usually enters the tube. There is usually little or no pain produced by the introduction of the catheter, there being only a disagreeable tickling sensation.

Air may be forced through the tubes either from the lungs of the physician, through a rubber tube attached to the outer end of the catheter, or with the air-bag. The permeability of the tube, and the condition of moisture or dryness, is indicated by the ease with which the air passes along the tube, and the sound produced by the air-douche upon the drum, as it may sometimes be seen to bulge out at each inflation, while in others, from the want of force on the stream of air, or adhesions in the middle ear, the concavity of the drum is not changed. By means of the otoscope we may hear the air strike against the drum.

The prognosis in chronic aural catarrh may be considered as favorable, that is, most of the cases presenting themselves are either cured or considerably improved. We must take into consideration the duration of the trouble, the changes and adhesions that may have taken place in the drum and middle ear.

Troltsh says in reference to changes in the drum: "If the appearance of the membrana tympani indicates partial and circumscribed morbid changes, especially of an adhesive nature, and if it is of a whitish color, the result of treatment is more unfavorable than the other condition, that is, the age and general conditon of the patient, duration and degree of the affection, would indicate at the first glance." He also believes, "when large spots of calcareous degenerations appear on the drums the prognosis is unfavorable."

Politzer gives an unfavorable prognosis in cases where there are continuous noises in the ear, and even if there is an improvement in the hearing it will not be permanent.

Swartze says: "There remains nothing but the test of treatments. If a local treatment persisted in carefully and rationally for a period of from eight to fourteen days, has no effect either upon the hearing, or the unpleasant sensations of the patient, any further treatment will probobly be of no avail."

The *treatment* of chronic aural catarrh is usually local, application being made direct to the mucous membrane of the tube and cavity of the tympanum by means of the catheter and Politzer's air-bag.

In many cases where the trouble is not of long standing and is confined principally to the tube, the forcing in of air alone produces an immediate and marked improvement in the hearing. It acts mechanically in opening the tube, and breaking up adhesions in the cavity of the tympanum.

The medicinal agents used in the treatment of chronic aural catarrh are the various irritants and astringents, used both in solution and in the form of vapor.

Those most used are the vapor of hot water, chloride of ammonium, in vapor and in solution (10 to 40 and 60 grains to ounce) vapor of the carbonate of ammonium, iodine, iodide of potash, (10 to 60 grains to ounce) sulphate of zinc, (1 to 10) liquor potassæ, etc. The agents may readily be forced into the middle ear by means of the catheter and Politzer's air-bag. Not more than fifteen to thirty drops of a solution (always warm) should be used at one injection, and repeated if it does not enter the middle ear. The more irritating of them sometimes produces considerable burning pain for a little while. I have used most of these, and believe I have had better results from the chloride of ammonium, twenty to forty grains to the ounce, than anything else. Blisters in the region of the ear, I believe, are useless and only annoy the patient.

The pharynx almost always requires attention, as there is considerable pharyngitis. If there is much congestion solution of nitrate of silver forty to sixty grains to the ounce, may be applied, by the physician, every day or two, to the pharynx. Any part of it may be reached by a curved probang. Gargles, also, of sodium, in solution, alum, chloride of sodium, etc., are useful.

Some patients in addition to the local will require a general treatment with cod liver oil, iron, etc.

